# Regulation and Function of Matrix Metalloproteinase-13 in Cancer Progression and Metastasis

**DOI:** 10.3390/cancers14133263

**Published:** 2022-07-03

**Authors:** Shun Li, David Mark Pritchard, Lu-Gang Yu

**Affiliations:** 1 Department of Biochemistry and Systems Biology, Institute of Systems, Molecular and Integrative Biology, University of Liverpool, Liverpool L69 3BX, UK; shun.li@liverpool.ac.uk; 2 Department of Molecular and Clinical Cancer Medicine, Institute of Systems, Molecular and Integrative Biology, University of Liverpool, Liverpool L69 3BX, UK; dmpritch@liverpool.ac.uk

**Keywords:** MMP-13, tumour growth, cancer invasion, metastasis, angiogenesis

## Abstract

**Simple Summary:**

MMP-13 is an enzyme that can digest and disrupt the tissue structures surrounding epithelial cells that line the surface of many internal organs, as well as the tissue structures surrounding endothelial cells that line the surface of blood vessels. The production of MMP-13 is tightly controlled in physiological conditions but is increased in various cancers and plays multiple roles in tumour progression and metastasis. This review summarises the current understanding of the regulation of MMP-13 production and discusses the actions of MMP-13 in cancer progression and metastasis.

**Abstract:**

Matrix metalloproteinase-13 (MMP-13) is a member of the Matrix metalloproteinases (MMPs) family of endopeptidases. MMP-13 is produced in low amounts and is well-regulated during normal physiological conditions. Its expression and secretion are, however, increased in various cancers, where it plays multiple roles in tumour progression and metastasis. As an interstitial collagenase, MMP-13 can proteolytically cleave not only collagens I, II and III, but also a range of extracellular matrix proteins (ECMs). Its action causes ECM remodelling and often leads to the release of various sequestered growth and angiogenetic factors that promote tumour cell growth, invasion and angiogenesis. This review summarizes our current understanding of the regulation of MMP-13 expression and secretion and discusses the actions of MMP-13 in cancer progression and metastasis.

## 1. Introduction

Matrix metalloproteinases (MMPs) are a family of 28 (so far) zinc-dependent endopeptidases [1]. According to their substrate specificities, MMPs are divided into several subfamilies of collagenases (MMP-1, MMP-8, MMP-13 and MMP-18), matrilysins (MMP-7 and MMP-26), gelatinases (MMP-2 and MMP-9), stromelysins (MMP-3, MMP-10 and MMP-11), membrane-type MMPs (MT-MMPs), glycosylphosphatidylinositol-anchored MMPs (MMP-17 and MMP-25) and others (MMP-12, MMP19, MMP-20, MMP-21, MMP-22, MMP-23, MMP-27 and MMP-28) [2,3,4]. All MMP family members share a conventional structure of a catalytic domain and a pro-peptide domain. In all cases except MMP-7 and MMP-26, the catalytic domain of MMPs contains a zinc-binding motif HEXXHXXGXXH [5] and is linked to a hemopexin domain by a flexible hinge region. With the exception of MMP-23, whose cysteine residue is located in a different amino acid sequence [6], the MMP amino-terminal pro-peptide domain contains a consensus sequence PRCXXPD (also known as cysteine switch). MMPs are produced in low amounts, and this is well-regulated under normal physiological conditions by various factors, including endogenous MMP inhibitors and tissue inhibitors of MMPs (TIMPs) [7]. Some MMP family members are, however, overexpressed in pathological disorders, such as cancer [8,9,10]. They are considered to be the primary contributors to the degradation of extracellular matrix (ECM) in tumour cell invasion. MMP family members have the ability to cleave ECM molecules with a wide range of substrate specificities [11]. Most ECM components can be degraded by MMP-3, -7, -10 and -11, while other MMPs, such as MMP-1, -8 and -13, preferentially digest collagen I, II and III located near the cells.

MMP-13 is an interstitial collagenase (also known as collagenase 3) and is overexpressed in various cancers [12,13,14] and in cancer stromal cells [15]. As a collagenase, MMP-13 can cleave not only collagens I, II and III, but also a wide range of ECM components. The expression and secretion of MMP-13 are regulated at the transcriptional and cellular levels [14,16,17,18,19,20]. Considerable evidence has shown that MMP-13-mediated degradation and remodelling of ECM plays a very important role in cancer pathogenesis and metastasis.

## 2. MMP-13 Structure

**Figure 1:**
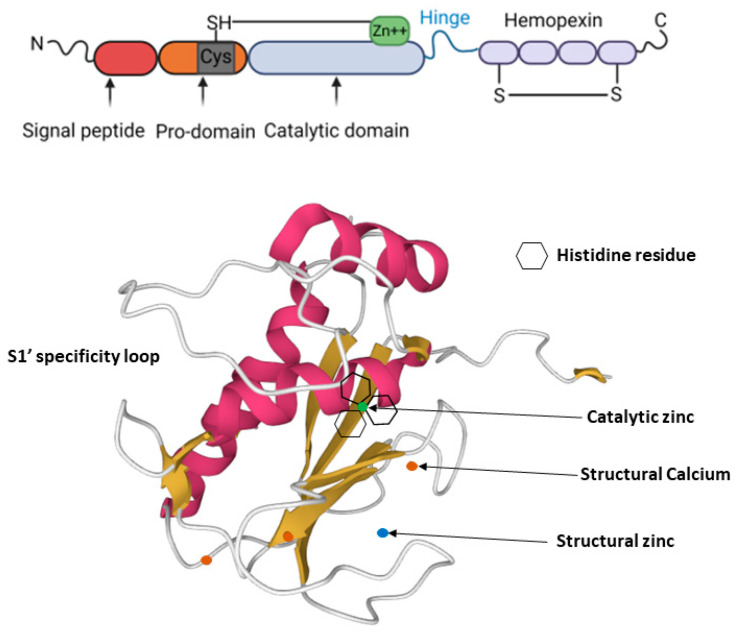
MMP-13 structure: MMP-13 consists of a highly conserved signal peptide, a pro-domain, a catalytic domain, a proline-rich hinge region and a C-terminal hemopexin-like domain (top panel). The cysteine residue (Cys 77) shown in grey within the pro-domain is linked to the catalytic zinc ion within the catalytic domain in pro-active MMP-13. The bottom panel shows the MMP-13 catalytic domain (PDB 2OW9). The structural zinc ion is in blue, the catalytic zinc ion is in green, and the three calcium ions are in orange. Three histidine residues, which bind to the catalytic zinc ion, are shown as hexagons.

MMP-13 is a 53kDa protein that consists of four domains, namely, the N-terminal signal sequence, basic pro-domain, catalytic domain and C-terminal hemopexin-like domain (Figure 1). Its C-terminal hemopexin-like domain is linked to the catalytic domain by a flexible hinge region. Its signal peptide domain controls the movement of the newly synthesized molecule and guides it to the endoplasm reticulum, while its pro-domain, which contains a zinc-interacting thiol (SH) group, keeps MMP-13 as an inactive zymogen form of pro-MMP-13. The catalytic domain of MMP-13 is shielded by the pro-domain in the inactive pro-MMP-13, and this prevents substrate access [21]. The MMP-13 catalytic domain, which is highly conserved among MMPs, includes three α-helixes and five β-sheets connected by eight loops [22]. The highly conserved catalytic domain of MMP-13, like other MMP members, has an extended zinc-binding motif, which consists of three zinc-binding histidines and a glutamate, a second structural zinc ion and three structural calcium ions, which are essential for enzyme stability [23,24]. The MMP-13 C-terminal hemopexin-like domain consists of four β-propeller elements and functions primarily for substrate specificity [25], as well as for degradation of triple-helical collagens [26]. Activation of pro-MMP-13 is carried out by other MMPs, such as MT1-MMP or MMP-2, on the cell surface [27], in which the cysteine residue is pulled out by conformational change to generate a functional active site, and this, in turn, enables enzymes to remove the pro-domain completely [28].

MMP-13 substrate specificity is largely controlled by its S pockets in the catalytic domain. There are multiple S pockets sitting on two sides of the catalytic zinc ion: (1) on the left side are pockets without a prime: S1, S2, S3…Sn; (2) on the right side are pockets with a prime: S1′, S2′, S3′… Sn’ [29]. The substances or inhibitors, in correspondence with the specific pockets, are named P1, P2…. Pn and P1′, P2′…Pn’, respectively [30]. It is believed that the S1′ pocket is the key contributor to establish MMP binding specificity, possibly because it is the most variable in depth among all the pockets [31]. While all MMPs contain the S1′ pocket, the volume and shape of each S1′ pocket varies [31]. MMP-13 possesses an exceptionally large S1′ pocket made of residues 245–253 [20]. However, given that MMP-13, MMP-8 and MMP-1 are all collagenases, but MMP-1 has only a small shallow pocket, the S1′ pocket of these MMP members may not be the only determining factor for their collagenase activity.

## 3. Regulation of MMP-13 Expression and Secretion

Due to its destructive nature as a protease towards a wide range of ECM proteins, MMP-13 was initially thought to be absent or to lack steady production in normal tissues [32,33]. However, subsequent studies revealed that MMP-13 is expressed in human chondrocytes and other healthy human connective tissues, such as cartilage and developing bone [34]. MMP-13 is also detected in normal epithelial and neuronal cells [35]. However, the expression and secretion of MMP-13 in normal human tissues are low and are tightly controlled at multiple levels by multiple factors.

The promoter of the human MMP-13 gene contains several binding sites for transcription factors. This includes a PEA-3 binding site and an AP-1 consensus sequence [36]. The combination of PEA-3/AP-1 acts as a responsive unit to growth factors, oncogenes and tumour promoters [36]. The human MMP-13 promoter also contains an osteoblast-specific element (OSE-2) binding site, ACCACA, which can be bound by transcription factor Cbfal [37]. The more distal region of the MMP-13 promoter also contains a Transforming growth factor-beta inhibitory element (TIE) binding site [38]. A conserved forkhead response element (FHRE) consensus sequence for FOXO3a has also been reported in the MMP-13 promoters in humans, mice and rats [39]. Although the precise mechanisms of MMP-13 regulation at the transcriptional level remain largely unknown, the presence of multiple bindings sites in its promoter for several transcription factors clearly indicates the importance of MMP-13 regulation at the transcriptional level. Indeed, several transcription factors have been reported to regulate MMP-13 expression. For example, the binding of ETS variant transcription factor 4 (ETV4) to the AP-1 binding site in the MMP-13 promoter region induced MMP-13 expression in breast cancer [40]. The binding of Small leucine zipper protein (sLZIP) to MMP-13 promoter increased MMP-13 expression in prostate cancer cells [41].

Various hormones, cytokines and growth factors regulate MMP-13 expression in human tissues. Interleukin-1 (IL-1), Interleukin-6 (IL-6) and Tumour necrosis factor alpha (TNF-α) can induce MMP-13 expression in primary chondrocytes [42]. This process is reported to involve nuclear translocation of nuclear factor kappa B (NF-κB) [43]. Growth factors, such as insulin-like growth factors (IGF)-I and -II, can inhibit MMP-13 expression in chondrocytes [44], while transforming growth factor-β1 (TGF-β1) has been shown to induce MMP-13 expression in human KMST fibroblasts [45].

**Figure 2:**
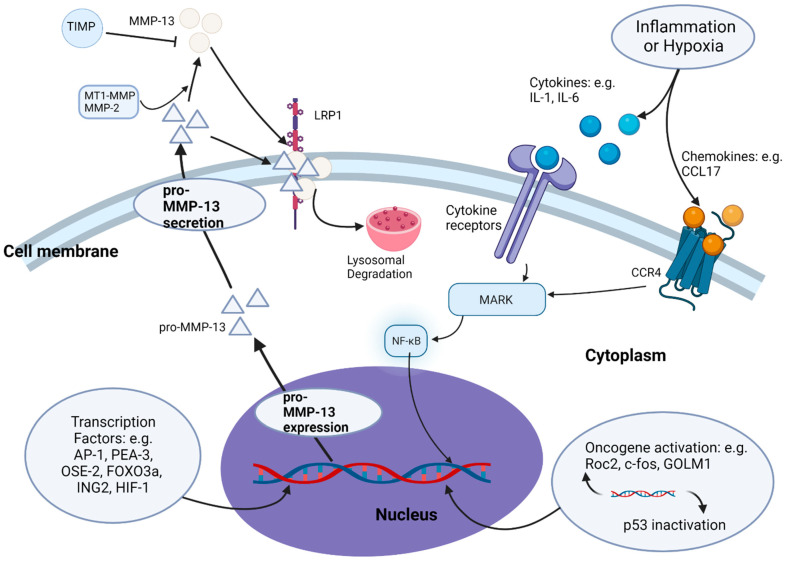
Regulation of MMP-13 expression and secretion in pathophysiology: MMP-13 expression and secretion in physiological conditions are tightly controlled by transcription factors and TIMPs. Various transcription factors bind directly to the MMP-13 promoter region. In cancer, the tight control of MMP-13 expression is disrupted by intrinsic and extrinsic mechanisms. Intrinsic mechanisms include changes of expression of oncogenes (e.g., Ror2) and tumour suppressor genes (e.g., p53). Extrinsic mechanisms include hypoxia and inflammation-induced secretion of cytokines and chemokines that activate downstream MARK signalling and NF-κB nuclear translocation to regulate MMP-13 expression. MMP-13 secretion is regulated by endocytosis through endocytic receptor LRP1 and subsequent degradation in lysosomes.

MMP-13 is normally secreted as an inactive pro-MMP-13 form by cells. Its activation is carried out through proteolytic cleavage of its pro-peptide domain by MT1-MMP and MMP-2 [27,46,47] (Figure 2). MMP-13 can also be activated by MMP-3 [27] and the major isoenzyme of human tumour-associated trypsinogen, trypsin-2 [48]. The activity of MMP-13 is controlled by TIMPs. Four TIMPs (TIMP1, TIMP2, TIMP3 and TIMP4) are known to exist in human tissues [49]. Each TIMP contains an N-terminal ‘wedge-shaped’ ridge domain, which binds to the MMP’s active site, and a C-terminal hemopexin interaction domain [50]. The function of TIMPs is to block substrate access to MMPs. In addition to the tight control of its expression and activity in normal physiological conditions, the secretion of MMP-13 to the outside of cells is also regulated by endocytosis. Low-density lipoprotein receptor-related protein 1 (LRP1) can bind to secreted MMP-13 (both pro- and activated forms) through its hemopexin domain and induce MMP-13 endocytosis and subsequent degradation in lysosomes in healthy human chondrocytes [34].

In cancer, the tight control of MMP-13 production and activity is disrupted by intrinsic or extrinsic mechanisms. The intrinsic mechanisms include changes of expression of oncogenes (e.g., Ror2) or proto-oncogenes (e.g., c-fos) and tumour suppressor genes (e.g., p53), which directly activate MMP-13 expression [51]. Suppression of oncogene Ror2 expression downregulated MMP-13 expression in osteosarcoma SaOS-2 cells [52]. Suppression of oncogene Golgi membrane protein 1 (GOLM1) inhibited MMP-13 expression in breast cancer [53], while suppression of tumour suppressor p53 increased MMP-13 expression in squamous cell carcinomas [54]. The extrinsic mechanisms involved in MMP-13 regulation include hypoxia and inflammation. The hypoxic microenvironment inside a tumour, created by a restricted oxygen supply from the increasing tumour size, induces cell necrosis [55]. This triggers inflammation and attracts leukocytes to the area to produce cytokines such as IL-1, IL-6 and TNF-α [56]. As discussed above, cytokines such as IL-1, IL-6 and TNF-α, β are important MMP-13 expression enhancers [42,51,57]. The secretion of these cytokines leads to an increase in MMP-13 expression [58,59,60]. Tumour hypoxia can also trigger the expression of hypoxia-inducible transcription factor (HIF)-1, which directly promotes MMP-13 gene expression by binding to the MMP-13 promotor or indirectly through promotion of the expression of the growth factors or cytokines [61] that regulate MMP-13 expression.

Recent studies have also reported the regulation of MMP-13 expression in cancer by other effectors, such as chemokines and endogenous enzymes. The binding of chemokine CCL17 to its receptor CCR4 was shown to enhance MMP-13 expression in bladder cancer through the activation of extracellular signal-regulated kinase (ERK) 1/2 signalling [62], and in colorectal cancer through the activation of ERK/NF-κB signalling [63]. The upregulation of ERK/NF-κB signalling enhanced the binding of NF-κB to Inhibitor of growth 2 (ING2) promoter, leading to the activation of ING2, which subsequently increased MMP-13 expression in colon cancer [64]. Small ubiquitin related modifier (SUMO)-specific protease 2 (SENP2) altered SUMOylation of the MMP-13 promoter and enhanced MMP-13 expression in bladder cancer [65]. FTO, a demethylase for N6-methyladenosine modification, was also shown to upregulate MMP-13 expression in oesophageal squamous cell carcinoma [66]. Overall, the expression, secretion and activation of MMP-13 in cancer is regulated at multiple levels and by many molecules, including cytokines, growth factors and proteases.

## 4. MMP-13 Expression in Cancer

Given its powerful and destructive action toward ECM, it is not surprising that higher MMP-13 expression frequently occurs in cancer. MMP-13 overexpression in cancer was first reported in breast cancer [12] and has subsequently been observed in many other cancers, such as colorectal [67,68], prostate [69], oesophageal [70,71], thyroid [72] and gastric cancers [73], as well as multiple myeloma (MM) [74] (Table 1). Over 50% higher MMP-13 expression is seen in bladder and non-small cell lung cancers [75], particularly at the invading front of the tumours [14]. A high level of MMP-13 expression was not only detected in primary breast cancers but also in metastatic lymph nodes that are associated with cancer aggressiveness [13,76]. Moreover, even higher MMP-13 expression is seen in the stromal cells surrounding the tumour [15]. Aggressive cancers have been shown to express higher levels of MMP-13 than less aggressive ones in prostate [77], breast [78] and head and neck cancers (HNSCC) [14,79]. Higher MMP-13 expression is also associated with lymph node metastasis and poor prognosis in bladder and non-small cell lung cancers [75,80] and with poorer patient survival in breast, prostate and head and neck cancers [14,78,79]. Overall, MMP-13 overexpression occurs in various cancers, including many common cancer types, and is often associated with tumour aggressiveness, poorer prognosis and reduced patient survival.

## 5. MMP-13 in Tumour Growth

The ECM contains multiple complex macromolecule components, such as collagens, proteoglycans and glycoproteins, and provides the scaffold support to tissues and organs [83]. ECM also helps to create an adequate environment for cell adhesion and tissue development. ECM generally consists of an architecture of fibrous polymers (e.g., collagens, elastins and resilins) [84] embedded in an undefined-shaped mixture of nonfibrous components (e.g., proteoglycans) [85]. ECM also includes basement membranes that are comprised of glycoproteins, such as laminin, fibronectin and entactin [86,87]. ECM component proteins are large multifunctional molecules with multiple domains. These domains are responsible for various functions, such as molecular recognition by cell surface receptors, predisposition to oligomerize and recognition by MMPs [88]. ECM serves as a general reservoir for growth factors and normally sequesters them in non-bioavailable forms. It also provides binding sites to cell surface adhesion molecules, such as integrins, for cell attachment and adhesion [89]. ECM degradation by MMPs such as MMP-13 releases sequestered growth factors, such as fibroblast growth factors (FGF) and TGF, which aid tumour cell proliferation [90]. MMP-mediated ECM degradation can also reveal the survival-associated hidden binding sites on ECM to enable ECM interaction with integrins on the tumour cell surface [91]. Although MMP-13 as a protease predominately degrades types I, II and III collagens, it can also cleave a range of other ECM components, such as gelatins [92], large tenascin C, fibronectin [93], aggrecan [94], fibrillin-1 [95], osteonectin [96] and perlecan. Increased expression of MMP-13 in tumour or stromal cells alters the collagen concentration in ECM and creates a more favourable environment for tumour growth [97]. MMP-13 can also deactivate non-matrix proteins, such as MCP-3 and SDF-1, by proteolytic actions [98,99,100] and reduce immune cell infiltration into the tumour and promote tumour growth [101].

Overexpression of MMP-13 mediated by GOLM1, C1r and Leptin has been shown to increase tumour growth in breast cancer [53], cutaneous squamous cell carcinoma [102] and pancreatic cancer [103]. The inhibition of MMP-13 expression by hammerhead ribozyme suppresses squamous cell carcinoma tumour growth and reduces the number of proliferating cells within the tumours [104]. Oral administration of an MMP-13 inhibitor, CMPD-1, twice a day, markedly delayed the growth of breast tumours in syngeneic mice [105]. Suppression of MMP-13 expression by antisense ribozyme reduced squamous cell carcinoma growth and led to the inhibition of cell invasion and induction of cell apoptosis [104]. The inhibition of MMP-13 expression by interferon gamma (IFN-γ) via activation of ERK1,2 and STAT1 in human cutaneous SCC cells (UT-SCC-7) and Ras-transformed human epidermal keratinocytes (A-5 cells) reduced cell proliferation and induced apoptosis [106]. MMP-13 suppression, mediated by p53 in malignantly transformed squamous epithelial cells, displayed an initial anti-invasive effect and was followed by induction of cell death [54]. Together, these studies indicate that overexpression of MMP-13 in cancer, either by tumour cells or stromal cells in the tumour microenvironment, makes important contributions to tumour growth.

## 6. MMP-13 in Cancer Cell Invasion and Metastasis

Tumour cell infiltration into ECM is a critical early step during cancer invasion and metastasis. Degradation of ECM by proteases such as MMPs creates the pathway for tumour cell infiltration and plays a key role in this process. Each MMP family member has its own substrate specificities towards ECM components. For example, MMP-1 targets primarily collagen III, while MMP-3 and -10 preferentially degrade proteoglycans, fibronectin and laminin [107]. MMP-13 has a relatively broad target specificity and can degrade collagens I, II and III, as well as a range of other ECM components [93,94,95,108]. For example, MMP-13 can cleave ECM component Laminin-5, which is mostly expressed in the basement membrane and is responsible for static adhesion of the epidermis and dermis for hemidesmosome formation [109,110,111]. Laminin-5 cleavage can reveal cryptic sites and increase mobility of epithelial cancer cells in tumour cell invasion and tissue remodelling [112,113,114]. MMP-13 is also involved in the activation of other MMPs, such as MMP-2 and MMP-9, by cleavage of the inactive pro-MMP-2 and pro-MMP-9 forms [19,115]. Proteolytic activation of MMP-9 by MMP-13 occurs in osteoarthritic chondrocytes [116] and chronic periodontitis [117]. It is possible that such an MMP-13/MMP-9 activation cascade may also exist in cancer.

MMP-13 also contributes to Epithelial-to-mesenchymal transition (EMT) in the tumorigenesis of epithelial cancer [118]. EMT confers epithelial cells with the metastatic properties of increased mobility and invasion, as well as an ability to escape apoptosis [119]. A primary EMT inducer is TGF-β [120]. The release of active TGF-β is normally carried out through proteolytical cleavage of the TGF-β-complex by MMP-28 [121]. Similar TGF-β activation has been reported by MMP-13 with chondrocytes in matrix vesicles, where secreted MMP-13 activated latent TGF-β in the progress of mineralization of growth plate cartilage [122]. The inhibition of MMP-13 expression in breast cancer cells at the tumour–bone interface significantly reduced TGF-β signalling, leading to a decrease in tumour-induced osteolysis [123].

MMP-13 also participates in the process of tumour cell infiltration into the blood or lymphatic vessels during metastasis. The blood capillaries are composed of an endoluminal side formed by endothelial cells and an abluminal side containing a basement membrane and vascular smooth muscle cells [124]. MMPs assist tumour cell penetration into blood capillaries by degrading the vascular basement membrane. MMP-9 is the primary contributor to vascular basement membrane degradation [125]. As MMP-13 is capable of activating MMP-9 by cleavage of the inactive pro-MMP-9 form [115,126,127], its action on MMP-9 activation can therefore promote tumour cell infiltration into the blood/lymphatic vessels at primary tumour sites, as well as the extravasation of invaded tumour cells from blood/lymphatic vessels at remote organs. The discovery that the inhibition of MMP-13 expression in MC38 colon cancer cells decreased the number of tumour cells extravasated from the hepatic vasculature in an experimental metastasis model is in line with this possibility [128].

Bone is one of the preferential metastasis sites of cancers such as breast cancer [129]. The bone ECM is rich with type I collagens [130]. MMP-13 is believed to be the primary protease to degrade type I collagen and aids breast cancer bone metastasis [131,132]. A higher MMP-13 level occurs at the tumour–bone interface of breast cancer [133]. Soluble factors, such as IL-6, produced by breast cancer cells induce MMP-13 expression in osteoblasts [134]. Tumour cells also produce parathyroid hormone-related protein (PTHrP) to induce MMP-13 expression through the activation of protein kinase C (PKC)-ERK1/2 signalling [135]. Inflammatory cells or osteoblasts could also produce PTHrP to stimulate MMP-13 secretion to promote breast cancer bone metastasis [136]. MMP-13 is also detected at the MM and bone marrow interface, and its presence is shown to promote MM cell bone marrow infiltration [137] and induce osteoclast [138]. Injection of MMP-13-selective inhibitor Zn^2+^-chelating compound, which targets the catalytic domain of MMP-13 [139], significantly reduced the level of bone destruction and delayed MM growth in an immunocompetent syngeneic mouse model with multiple myeloma [74].

The arrival of tumour cells at distant organs, which is an alien microenvironment from the primary tumour sites, is often unfavourable for tumour cells to survive and grow. This can lead the tumour cell to die from apoptosis or enter a ‘silent’ mode without proliferation or death. The successful establishment of a metastasis at the distant organs/sites requires the build-up of a permissive environment, known as a pre-metastatic niche. Pre-metastatic niche formation is driven by many factors, including primary tumour-derived factors, tumour-mobilized bone marrow-derived cells (BMDC), hypoxia, ECM remodelling and exosomes [140]. The formation of a pre-metastatic niche is triggered by the release from the primary tumour of factors such as growth factors (e.g., TGF-α and -β) or cytokines (e.g., TNF-α). These, in turn, induce the expression and secretion of chemoattractants (e.g., S100 proteins) by the endothelium [141] and the production of fibronectin by fibroblasts at the niche site [142]. The expression of S100 can lead to the activation of NF-κB signalling [143] and the subsequent production of MMPs, including MMP-13, by stromal cells [144]. The structure of the new site’s intrinsic ECM is often less ideal for attachment, metabolism and migration of the recruited BMDC and immune cells. The production and action of the new MMPs, including MMP-13, cause ECM remodelling and aid the formation of the pre-metastatic niche. BMDCs and several immune cells are also recruited to the site to assist in the establishment of the pre-metastatic niche. These immune cells secrete inflammatory cytokines, growth factors and proangiogenic molecules to create a favourable local microenvironment for the extravasated tumour cells [145]. MMP-9 is responsible for the recruitment of BMDCs to the niche site via releasing soluble factors, such as Kit-ligand from BM stromal cells [146], and can be activated by MMP-13 through proteolytic cleavage of pro-MMP-9.

Lysl Oxidase (LOX) is another key regulator involved in the recruitment of BMDCs and is often released by primary tumours during hypoxia [147]. LOX cross-links collagen IV in the basement membrane and allows CD11b+ myeloid cells to adhere and release MMPs at the niche site [147]. The release of these MMPs further degrades collagen fibres and releases collagen IV peptides, which act as chemoattractants to aid recruitment of BMDCs to the niche site. As the inhibition of LOX by licofelone can reduce MMP-13 expression in human osteoarthritic chondrocytes [148], LOX-mediated MMP-13 expression can therefore contribute to pre-metastatic niche formation by aiding BMDC recruitment to niche sites.

Exosomes are small vesicles that contain proteins, mRNAs, microRNAs, small RNAs and DNA fragments [149]. Tumour-associated exosomes can assist pre-metastatic niche formation and aid tumour cell communication by transportation of regulatory molecules [150,151]. MMP-13 occurs in primary tumour cell-derived exosomes under hypoxic conditions [152]. It is later released into the circulation to modulate ECM components and helps the establishment of pre-metastasis sites [152]. Overall, MMP-13 makes important contributions to tumour cell invasion at primary tumour sites, as well as the establishment of tumour cells at remote organs in metastasis through the degradation and remodelling of ECM and the activation of other MMPs.

## 7. MMP-13 in Angiogenesis

Angiogenesis is an important process during cancer pathogenesis. It provides essential nutrients and a blood supply for sustained tumour growth and development [153]. The multiple-stepped process of angiogenesis consists of: (1) degradation of basement membrane and ECM around the blood vessels; (2) activation of endothelial cells for migration and proliferation; (3) transformation of endothelial cells into capillary tubes [154]. Angiogenesis is tightly regulated in normal tissues, but this tight control is disrupted in cancer [155] by a group of angiogenic factors released by endothelial cells, tumour cells, stromal cells and ECM [156,157,158,159,160]. These angiogenic factors can be either pro- or anti-angiogenic [161]. Pro-angiogenetic regulators include vascular endothelial growth factor (VEGF), basic fibroblast growth factor (bFGF), TGF-α and -β, epidermal growth factor (EGF), platelet-derived growth factor (PDGF), placental-derived growth factor and angiopoietin -1 and -2 [162], while anti-angiogenic regulators include angiostatin, endostatin, tumstatin, platelet factor-4, interleukin-12, thrombospondin-1, TIMPs and interferon-α, -β and -γ [154].

Many MMP family members are known to take part in the process of angiogenesis. Their primary action is to degrade ECM components. This leads to the release of ECM-bound angiogenic factors that enable endothelial cells to invade the tumour stroma leading to new blood vessel formation [163]. Interstitial collagens are the major proteins in the vascular tissue milieu [164]. The structural triple-helical, fibrillar collagen in vascular tissue is highly resistant to many proteolytic enzymes, such as trypsin, plasmin, extracellular serine proteases and many MMP members [165]. Only a limited number of MMPs can cleave the highly structured fibrillar collagens. MMP-2 and -9 can cleave fibrillar type I collagen and release ECM-bound angiogenic growth factors [88] to aid blood vessel formation and endothelial cell invasion [166,167]. MMP-13 is one of the proteases that can digest well-structured fibrillar collagens in ECM around the blood vessel and contribute to the release of ECM-bound angiogenic regulators [168]. MMP-13 has been shown to efficiently and specifically cleave interstitial collagens to initiate ECM remodelling and promote new blood vessel formation in the chorioallantoic membrane in a chicken embryo model [164]. MMP-13 expression in stromal fibroblasts was shown to enhance VEGF and VEGFR-2 concentrations in the tumour cell invasive areas around blood vessels and promote angiogenesis in skin carcinoma [169]. MMP-13-mediated release of VEGF-C increased cancer cell spreading through lymphatic vascular systems in paediatric multiple myeloma [170]. Higher MMP-13 expression correlates closely with a higher number of blood vessels in human head and neck cancer [168]. Overall, the expression and presence of MMP-13 in cancer and stromal cells is actively involved in promoting angiogenesis in tumour cell metastatic spreading.

## 8. Concluding Remarks

**Figure 3:**
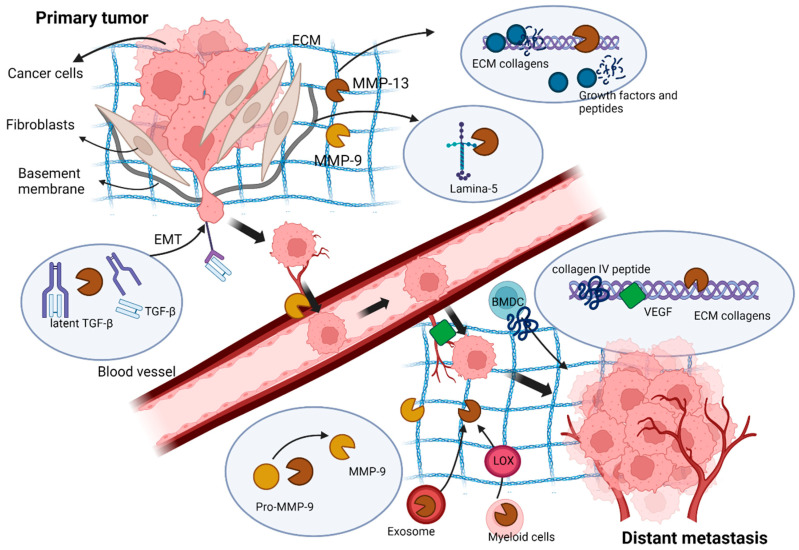
The action of MMP-13 in cancer progression and metastasis: MMP-13, produced by cancer cells and stromal fibroblasts, cleaves ECM and leads to ECM remodelling and liberation of ECM-bound growth factors and angiogenic factors that promote tumour cell proliferation, invasion, EMT and angiogenesis at primary tumour sites. At distant metastasis sites, MMP-13-mediated ECM remodelling and release of growth factors and collagen IV peptides help the recruitment of immune cells to the pre-metastatic niche and the establishment of a favourable metastasis environment.

As an interstitial collagenase that can cleave not only collagens but also a range of other ECM components, MMP-13 is overexpressed in various cancers and plays multiple roles in cancer development, progression and metastasis. Its proteolytic action leads to ECM remodelling and release of ECM-sequestered growth factors, cytokines and angiogenic factors that promote tumour cell proliferation, EMT, invasion and angiogenesis. MMP-13-mediated ECM remodelling and release of growth factors also aids the recruitment of immune cells to the pre-metastatic niche and helps the establishment of secondary metastasis sites in distant organs (Figure 3). Despite the critical involvement of MMP-13 in cancer progression and metastasis, the precise mechanisms of its regulation and actions are still not fully understood. Much also remains unknown about the possible coordination of MMP-13 action with other MMP family members during cancer pathogenesis. Little is known about whether MMP-13 appearance/overexpression in cancer and stromal cells influences the activity or function of cell surface molecules, such as cell adhesion and signalling proteins. Future research will help to gain further insights into the role and actions of MMP-13 in cancer and determine whether MMP-13 represents an effective therapeutic target for this disease.

## Figures and Tables

**Table 1:** MMP-13 expression in common cancer and its function and clinical significance.

Cancer Type	Expression Level	Function and Clinical Significance	References
Breast cancer	Increased	Increased tumour growth, invasion and metastasis; potential diagnostic biomarker	[13]
Prostate cancer	Increased	Increased tumour differentiation, invasion and metastasis; diagnostic biomarker; poor prognosis	[69,81,82]
Bladder cancer	Increased	Increased tumour invasion and metastasis; poor prognosis	[75]
Colorectal cancer	Increased	Increased tumour growth, invasion and metastasis; poor prognosis	[47,68]
Oesophageal cancer	Increased	Promoted cancer aggressiveness; poor prognosis	[70,71]
Head and neck cancer	Increased	Increased tumour invasion and metastasis; poor prognosis	[14]
Lung cancer	Increased	Promoted lymph node metastasis; poor survival	[80]
Oesophageal cancer	Increased	Promoted cancer aggressiveness; poor prognosis	[70]
Gastric cancer	Increased	Increased tumour invasion and metastasis; poor prognosis	[73]
Thyroid cancer	Increased	Increased tumour invasion and metastasis; poor prognosis	[72]
Multiple Myeloma	Increased	Promoted tumour growth and MM-induced osteolysis; poor prognosis	[74]

## Abbreviations

bFGF	Basic fibroblast growth factor
BMDC	Bone marrow-derived cells
ECM	Extracellular matrix
EGF	Epidermal growth factor
EMT	Epithelial-to-mesenchymal transition
ER	Extracellular signal-regulated kinase
ETV	ETS variant transcription factor 4
FAK	Focal adhesion kinase
FHRE	Forkhead response element
FGF	Fibroblast growth factors
GOLM1	Golgi membrane protein 1
HIF	Hypoxia-inducible transcription factor
HNSCC	Squamous cell carcinoma of the head and neck
IL	Interleukin
IFN-γ	Interferon gamma
ING2	Inhibitor of growth 2
IGF	Insulin-like growth factor
IFN-β	Interferon β
LOX	Lysl Oxidase
MMP	Matrix metalloproteinase
NF-κB	Nuclear factor kappa B
MM	Multiple Myeloma
OSE-2	Osteoblast-specific element
PDGF	Platelet-derived growth factor
PTHrP	parathyroid hormone-related protein
PKC	Protein kinase C
SH	Zinc-interacting thiol
SUMO	Small ubiquitin related modifier
SENP2	SUMO-specific protease 2
TGF-β	Transforming growth factor-β1
TIMPs	Tissue inhibitors of MMPs
TIE	Transforming growth factor-beta inhibitory element
TNF-α	Tumour necrosis factor alpha
VEGF	Vascular endothelial growth factor
